# A Reference Hand-Book of the Medical Sciences

**Published:** 1902-09

**Authors:** 


A REFERENCE HAND-BOOK OF THE MEDICAL SCIENCES, embracing the entire range of scientific and practical medicine and allied sciences. By various writers. A new edition, completely revised and rewritten. Edited by Albert H. Buck, M. D., of New York City. Volume IV. Illustrated by chromolithographs and eight hundred and fifty-nine half-tone and wood engravings. Pp., 873. Price $—— per volume.

To any one acquainted with the first edition of the Reference Hand-book, no word of commendation is necessary. It occupies the best space in the doctor's library. The subjects treated are all that pertain to medical science and allied sciences, making it invaluable as a work of reference—a whole library within itself. The writer of this review was asked some time ago by a prominent minister for a reliable article on *suicide*, and was surprised that he did not have a discussion of the subject in a number of other books in his library. Out of seven or eight hundred volumes the Reference Hand-book alone had the desired article. And so it is the Reference Hand-book covers the field. T. J. B.

